# The m^6^A methyltransferase METTL16 negatively regulates MCP1 expression in mesenchymal stem cells during monocyte recruitment

**DOI:** 10.1172/jci.insight.162436

**Published:** 2023-03-22

**Authors:** Zhaoqiang Zhang, Zhongyu Xie, Jiajie Lin, Zehang Sun, Zhikun Li, Wenhui Yu, Yipeng Zeng, Guiwen Ye, Jinteng Li, Feng Ye, Zepeng Su, Yunshu Che, Peitao Xu, Chenying Zeng, Peng Wang, Yanfeng Wu, Huiyong Shen

**Affiliations:** 1 Department of Orthopedics, and; 2 Center for Biotherapy, The Eighth Affiliated Hospital, Sun Yat-sen University, Shenzhen, China.

**Keywords:** Immunology, Stem cells, Adult stem cells, Chemokines, Epigenetics

## Abstract

Mesenchymal stem cells (MSCs) possess strong immunoregulatory functions, one aspect of which is recruiting monocytes from peripheral vessels to local tissue by secreting monocyte chemoattractant protein 1 (MCP1). However, the regulatory mechanisms of MCP1 secretion in MSCs are still unclear. Recently, the N^6^-methyladenosine (m^6^A) modification was reported to be involved in the functional regulation of MSCs. In this study, we demonstrated that methyltransferase-like 16 (METTL16) negatively regulated MCP1 expression in MSCs through the m^6^A modification. Specifically, the expression of METTL16 in MSCs decreased gradually and was negatively correlated with the expression of MCP1 after coculture with monocytes. Knocking down METTL16 markedly enhanced MCP1 expression and the ability to recruit monocytes. Mechanistically, knocking down METTL16 decreased *MCP1* mRNA degradation, which was mediated by the m^6^A reader YTH N^6^-methyladenosine RNA-binding protein 2 (YTHDF2). We further revealed that YTHDF2 specifically recognized m^6^A sites on *MCP1* mRNA in the CDS region and thus negatively regulated MCP1 expression. Moreover, an in vivo assay showed that MSCs transfected with METTL16 siRNA showed greater ability to recruit monocytes. These findings reveal a potential mechanism by which the m^6^A methylase METTL16 regulates MCP1 expression through YTHDF2-mediated mRNA degradation and suggest a potential strategy to manipulate MCP1 expression in MSCs.

## Graphical abstract

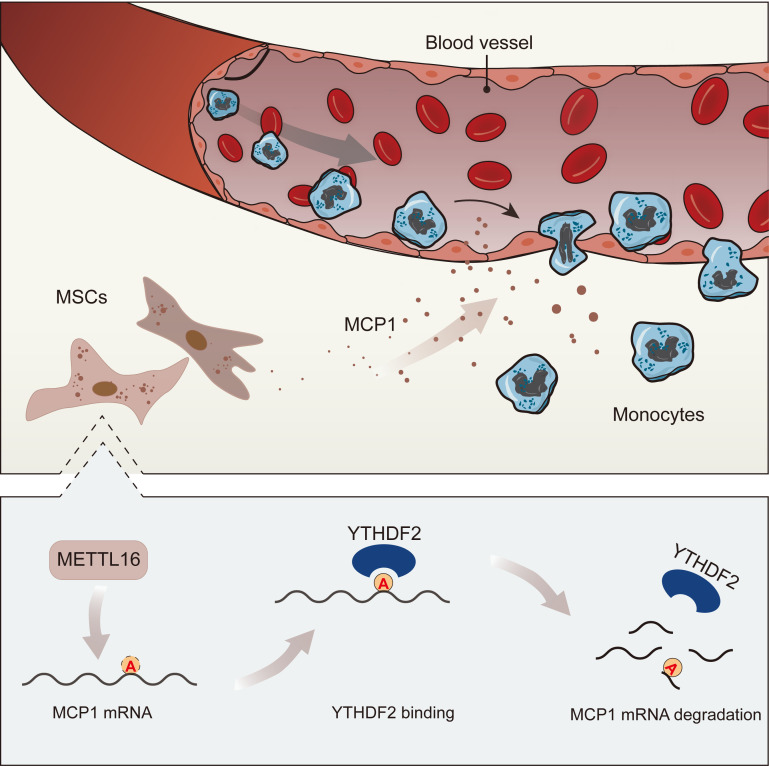

## Introduction

Mesenchymal stem cells (MSCs) are a subset of pluripotent stromal stem cells characterized by various functions in vivo (1), the most important of which is the strong immunoregulatory effects on both innate and adaptive immune responses in various tissues (2). Through cell-cell contact and the secretion of cytokines, MSCs exert their powerful immunoregulatory effects on immune cells (3). Monocytes are important immune cells that are recruited by MSCs from peripheral blood into inflamed tissues and then differentiate into M1 inflammatory or M2 antiinflammatory macrophages. This process is essential for immune homeostasis (4), and MSC-induced monocyte recruitment is involved in many diseases, including infection (5), atherosclerosis (6), and liver fibrosis (7). Thus, it is essential to ascertain the mechanisms by which MSCs recruit monocytes, which remain unclear.

Monocyte chemoattractant protein 1 (MCP1), also known as CCL2, is one of the main chemokines that regulate monocyte recruitment in inflammation and infection (8). By secreting a large amount of MCP1, MSCs recruit monocytes and regulate the immune response in many biological processes and diseases, including tissue injury (9), wound healing (10), and inflammatory diseases (11). Previously (12), we found that abnormal MCP1 secretion from MSCs and monocyte infiltration intensified the development of chronic inflammation in patients with ankylosing spondylitis. Moreover, modulating MCP1 expression in MSCs to treat diseases has been reported and is considered a prospective strategy (13). Exactly how MSCs manipulate MCP1 secretion in the local environment remains poorly understood and needs further study.

Dynamic RNA modifications have been recently revealed as an important regulatory mechanism of gene expression (14). N^6^-methyladenosine (m^6^A) methylation is one of most common internal modifications of mRNA and was found to regulate mRNA stability and precursor mRNA processing in mammalian cells (15). Attention has been given to the biological functions of m^6^A modifications in immune regulatory processes in recent years (16). Recently, a study indicated that the m^6^A modification may also participate in MCP1 expression (17). Moreover, our studies showed that m^6^A modifications could regulate the directional migration of MSCs and thus aggravate chronic inflammation in ankylosing spondylitis (18). Whether m^6^A modifications are involved in MCP1 regulation in MSCs is relatively unreported and poorly understood.

In this study, we investigated the role of the m^6^A modification in MCP1 expression and the subsequent monocyte recruitment ability of MSCs. Through this study, we demonstrated that the m^6^A methylase METTL16 negatively regulated MCP1 expression through mRNA degradation mediated by YTH N^6^-methyladenosine RNA-binding protein 2 (YTHDF2). This study may help to elucidate novel mechanisms of MCP1 secretion and monocyte recruitment mediated by MSCs in immune regulation and may also suggest a potential therapeutic strategy to manipulate MCP1 expression in MSCs in some disorders involving MCP1.

## Results

### MCP1 expression is negatively correlated with the m^6^A methylase METTL16 in MSCs.

**Figure 1:**
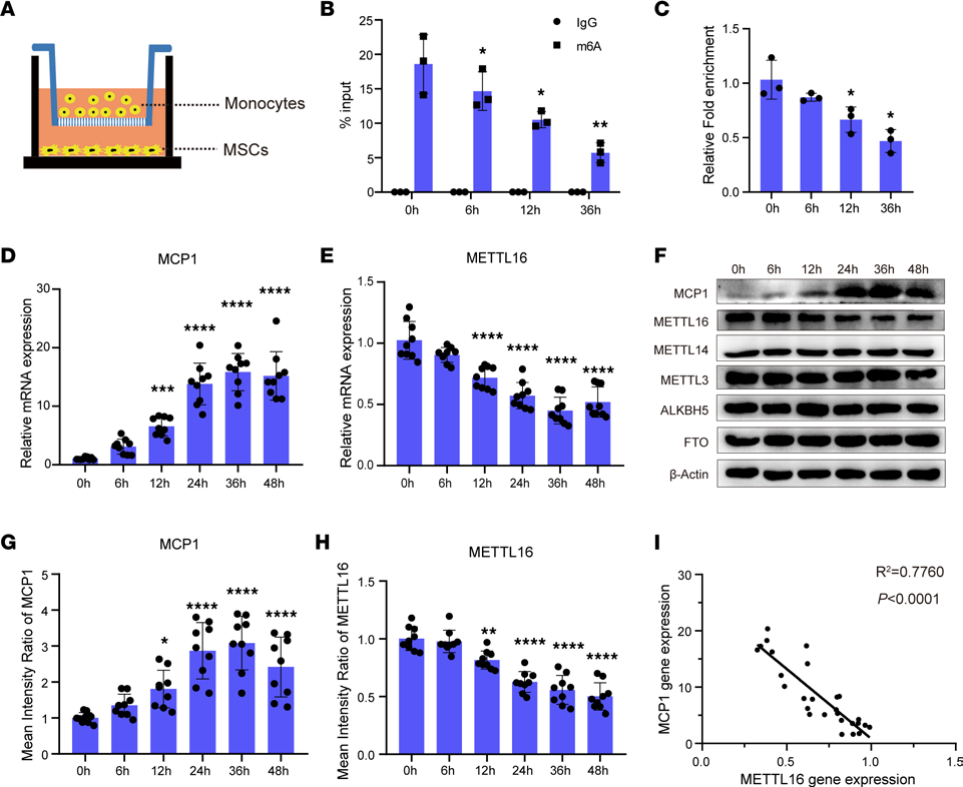
MCP1 expression is negatively correlated with the m^6^A methylase METTL16 in MSCs. (**A**) Schematic diagram of the MSC and monocyte coculture system. (**B** and **C**) m^6^A modification levels of *MCP1* mRNA of MSCs (*n* = 3) cultured without (0 hour) or with monocytes for 6 hours, 12 hours, and 36 hours. (**D** and **E**) Relative mRNA expression of *MCP1* and *METTL16* of MSCs (*n* = 9) cultured with monocytes at different time points. (**F**) Representative blot images of MCP1, METTL16, METTL14, METTL3, ALKBH5, and FTO of MSCs (*n* = 9) cultured with monocytes at different time points. (**G** and **H**) The mean intensity ratio of MCP1 and METTL16 of MSCs (*n* = 9) cultured with monocytes at different time points. (**I**) The correlation between *MCP1* mRNA and *METTL16* mRNA expression in the MSCs cocultured with monocytes (*R*^2^ = 0.7760, *P* < 0.0001). Data are presented as the mean ± SD. One-way ANOVA followed by Bonferroni’s test was performed by comparison with the 0-hour group (**B**–**E**, **G**, and **H**). *P < 0.05, **P < 0.01, ***P < 0.001, ****P < 0.0001. MSCs, mesenchymal stem cells; MCP1, monocyte chemoattractant protein 1.

To simulate the intercellular crosstalk between MSCs and monocytes, we cocultured MSCs with CD14^+^ monocytes in vitro using a Transwell system (Figure 1A). Before coculture (0 hours), m^6^A methylation on *MCP1* mRNA could be detected at a high level. When the coculture condition lasted for 6, 12, or 36 hours, the m^6^A methylation level of MCP1 was gradually decreased (Figure 1, B and C). Moreover, after coculture with monocytes, the *MCP1* mRNA expression of MSCs was substantially higher in MSCs cocultured with monocytes than in MSCs without cocultured monocytes, and gradually upregulated as the duration increased (Figure 1D). This expression pattern was also confirmed by Western blot analysis of the MCP1 protein level (Figure 1F).

We then investigated the expression levels of enzymes relevant to m^6^A methylation, including METTL3, METTL14, METTL16, and the m^6^A demethylases alkylation repair homolog protein 5 (ALKBH5) and fat mass and obesity-associated protein (FTO). Compared with that of MSCs without coculture, the mRNA expression of *METTL16* in cocultured MSCs was decreased and gradually downregulated as coculture time extended (Figure 1E), while the mRNA expression levels of *METTL3*, *METTL14*, *ALKBH5*, and *FTO* showed no significant changes when cocultured with monocytes (Supplemental Figure 1A; supplemental material available online with this article; https://doi.org/10.1172/jci.insight.162436DS1). Western blot analysis of the protein levels of these enzymes showed similar results (Figure 1, F–H, and Supplemental Figure 1B). We further analyzed the relationship between *MCP1* and *METTL16* mRNA expression in the coculture experiment, and the results revealed a strong correlation between *MCP1* and *METTL16*, with a coefficient of determination (*R*^2^) of 0.7760 (*P* < 0.0001) (Figure 1I).

### METTL16 negatively regulates MCP1 expression in MSCs and its monocyte recruitment capacity.

**Figure 2:**
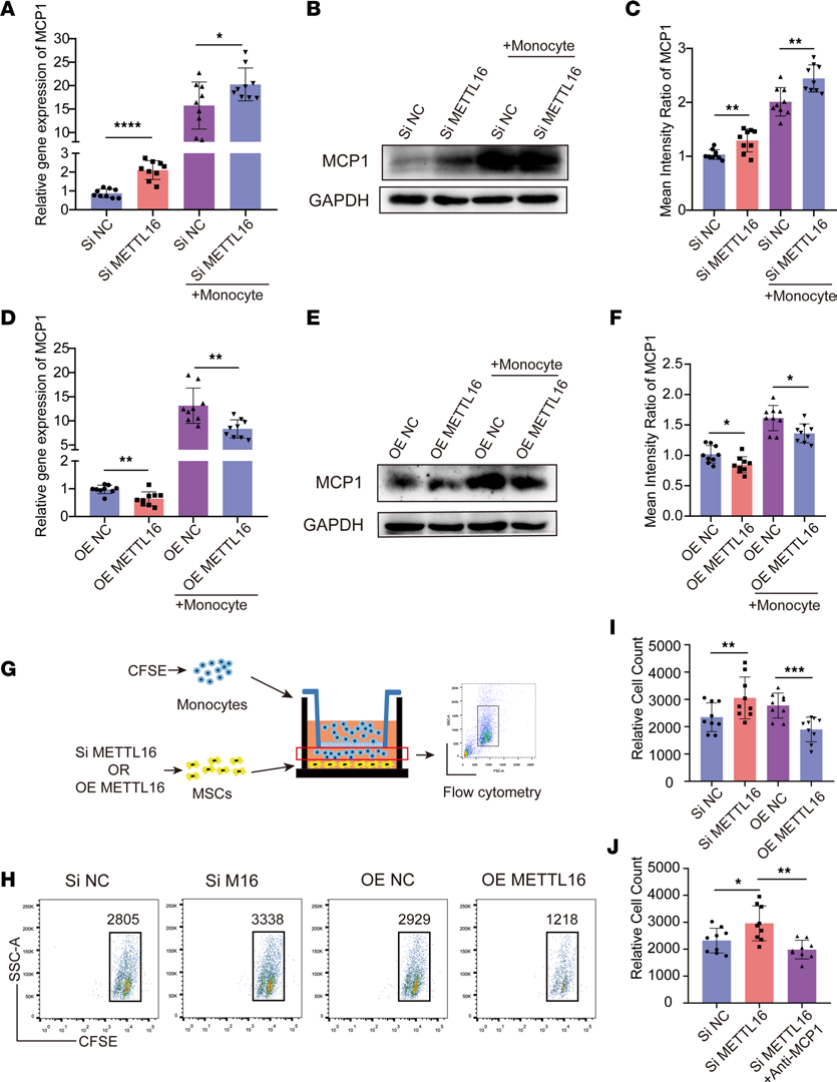
METTL16 negatively regulates MCP1 expression in MSCs and its monocyte recruitment capacity. (**A**) Relative *MCP1* mRNA expression in siNC- or siMETTL16-treated MSCs (*n* = 9) cultured with or without monocytes. (**B**) Representative blot images of MCP1 in siNC- or siMETTL16-treated MSCs (*n* = 9) cultured with or without monocytes. (**C**) The mean intensity ratio of MCP1 in siNC- or siMETTL16-treated MSCs (*n* = 9) cultured with or without monocytes. (**D**) Relative mRNA expression of *MCP1* in OE NC–treated or OE METTL16–treated MSCs (*n* = 9) cultured with or without monocytes. (**E**) Representative blot images of MCP1 in OE NC–treated or OE METTL16–treated MSCs (*n* = 9) cultured with or without monocytes. (**F**) The mean intensity ratio of MCP1 in OE NC– or OE METTL16–treated MSCs (*n* = 9) cultured with or without monocytes. (**G**) Schematic diagram of the monocyte recruitment system. (**H** and **I**) Representative flow cytometry histograms and relative cell count of monocytes recruited by MSCs (*n* = 9). (**J**) Relative cell count of monocytes recruited by siNC- or siMETTL16-treated MSCs (*n* = 9) with or without MCP1-neutralizing antibody. Data are presented as the mean ± SD. Two-tailed Student’s *t* test was performed in panels **A**, **C**, **D**, **F**, and **I** and 1-way ANOVA followed by Bonferroni’s test was performed in **J**. *P < 0.05; **P < 0.01; ***P < 0.001; ****P < 0.0001. MSCs, mesenchymal stem cells; MCP1, monocyte chemoattractant protein 1; siNC, control siRNA; siMETTL16, siRNA for METTL16; OE NC, control lentiviruses; OE METTL16, lentiviruses overexpressing METTL16.

To investigate the role of m^6^A in regulating MCP1 expression, we knocked down the m^6^A methylases METTL3, METTL14, and METTL16 and the m^6^A demethylases ALKBH5 and FTO by using an RNA interference method. Two specific siRNAs for each gene were designed, and the knockdown efficiency of each siRNA was confirmed at both the RNA and protein levels of the target genes. As shown by the quantitative real-time PCR (qPCR) and Western blot results, siMETTL3-2, siMETTL14-1, siMETTL16-2, siALKBH5-2, and siFTO-1 showed better efficiency and were chosen for the following experiments (Supplemental Figure 3, A and B). RNA interference was performed on MSCs with or without monocyte coculture. After knockdown of the expression of METTL16, the qPCR results showed that *MCP1* expression in MSCs without monocyte coculture was increased approximately 2-fold compared with that in the control group. When MSCs were cocultured with monocytes after knocking down METTL16, the expression of *MCP1* also showed an increase compared with that in the corresponding control group (Figure 2A). Knocking down METTL3, METTL14, FTO, and ALKBH5 in MSCs did not significantly affect the mRNA expression of *MCP1* under the coculture conditions (Supplemental Figure 2, A–D). Western blot analysis also showed a similar result; knocking down METTL16 increased MCP1 expression both in coculture and in the absence of coculture conditions (Figure 2, B and C). The Western blot results of MCP1 in the MSCs with METTL3, METTL14, FTO, and ALKBH5 knockdown were similar to the qPCR results (Supplemental Figure 2, E–H). We then overexpressed METTL16 in MSCs. The overexpression efficiency of lentiviral METTL16 was assessed at both the mRNA and protein levels (Supplemental Figure 3, C and D). The data showed that overexpression of METTL16 decreased the mRNA and protein levels of MCP1 in the MSCs with or without coculture conditions (Figure 2, D–F). Collectively, these data suggest that METTL16 can negatively regulate MCP1 expression in MSCs.

To verify whether METTL16 expression affects the ability of MSCs to recruit monocytes, we used a Transwell system with a 5 μm pore size, and MSCs with METTL16 knockdown or overexpression were seeded in the lower chamber (Figure 2G). Flow cytometry analysis revealed that the MSCs with METTL16 knockdown recruited more monocytes than the controls, while the MSCs overexpressing METTL16 recruited fewer monocytes than the corresponding controls (Figure 2, H and I). To further confirm that MCP1 secreted by MSCs was responsible for monocyte recruitment, we added an MCP1-neutralizing antibody to the culture medium in the lower chamber. Compared with the MSCs with METTL16 knockdown only, MSCs recruited fewer monocytes when there was an MCP1-neutralizing antibody in the medium (Figure 2J).

### METTL16 regulates MCP1 m^6^A modification and its mRNA stability.

**Figure 3:**
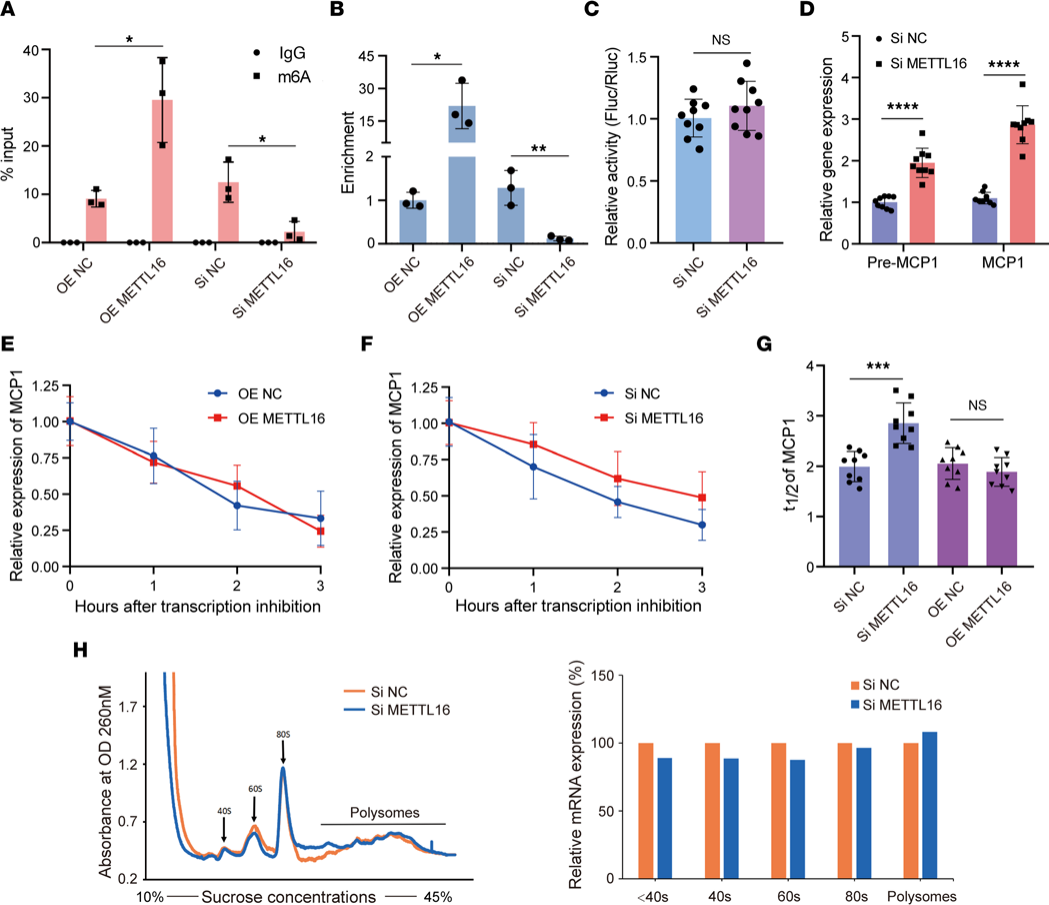
METTL16 regulates *MCP1* m^6^A modification and its mRNA stability. (**A** and **B**) m^6^A modification levels of *MCP1* mRNA in siNC-/siMETTL16- or OE NC–/OE METTL16–treated MSCs (*n* = 3). (**C**) *MCP1* promoter activity in siNC- or siMETTL16-treated MSCs (*n* = 9) expressed as the ratio of firefly versus Renilla luciferase (Fluc/Rluc). (**D**) Relative expression of *MCP1* precursor mRNA (pre-MCP1) and mature mRNA (MCP1) in siNC- or siMETTL16-treated MSCs (*n* = 9). (**E**) Degradation curves of *MCP1* mRNA in OE NC– or OE METTL16–treated MSCs (*n* = 9). (**F**) Degradation curves of *MCP1* mRNA in siNC- or siMETTL16-treated MSCs (*n* = 9). (**G**) The *t*_1/2_ analysis of *MCP1* mRNA in siNC-/siMETTL16- or OE NC–/OE METTL16–treated MSCs (*n* = 9). (**H**) The abundance of *MCP1* mRNA on polysomes was not changed significantly with knockdown of METTL16 (*n* = 9). Data are presented as the mean ± SD. Two-tailed Student’s *t* test was performed in panels **A**–**D** and **G**. *P < 0.05; **P < 0.01; ***P < 0.001; ****P < 0.0001. NS, not significant; MSCs, mesenchymal stem cells. MCP1, monocyte chemoattractant protein 1; siNC, control siRNA; siMETTL16, siRNA for METTL16; OE NC, control lentiviruses; OE METTL16, lentiviruses overexpressing METTL16.

As METTL16 is a key m^6^A methyltransferase, to further verify whether METTL16 is responsible for inducing m^6^A modifications on *MCP1* mRNA in MSCs, we overexpressed and knocked down METTL16 and then analyzed the m^6^A level on *MCP1* mRNA by m^6^A RNA immunoprecipitation–qPCR (RIP-qPCR) analysis. The results showed that the m^6^A modification levels of *MCP1* in the MSCs transfected with METTL16 overexpression lentiviruses were significantly higher than those in the MSCs transfected with control lentiviruses. Moreover, the m^6^A modification levels of *MCP1* were lower in the siMETTL16 group than the negative control siRNA (siNC) group (Figure 3, A and B), indicating that METTL16 could induce m^6^A modification of *MCP1* mRNA. Next, we further investigated how METTL16 regulates MCP1 expression. The *MCP1* promoter sequence was cloned into the luciferase reporter plasmid. However, there was no difference between the METTL16-knockdown MSCs and the control MSCs, suggesting that METTL16 did not affect the transcription of MCP1 (Figure 3C). Moreover, we tested both the expression of mature (*MCP1*) and precursor (pre-*MCP1*) mRNA of *MCP1*. Compared with that of the control MSCs, the expression of both pre-*MCP1* and *MCP1* mRNA in the METTL16-knockdown MSCs was enhanced significantly (Figure 3D). Then, we treated the METTL16-overexpressing or METTL16-knockdown MSCs with actinomycin D to inhibit transcription. The RNA stability assay showed that the half-life of *MCP1* mRNA in the METTL16-knockdown MSCs was significantly longer than that in the control MSCs (Figure 3, F and G). However, there was no significant difference in the half-life of *MCP1* mRNA between the METTL16-overexpressing MSCs and the relevant control MSCs (Figure 3, E and G). To explore whether METTL16 regulates the translation efficiency of MCP1, we performed a polysome profiling assay, and the results showed that the abundance of *MCP1* mRNA on polysomes was not changed with METTL16 knockdown (Figure 3H). Collectively, these results indicated that m^6^A-regulated MCP1 expression was related to the regulation of mRNA degradation.

### METTL16 accelerates MCP1 mRNA degradation through YTHDF2.

**Figure 4:**
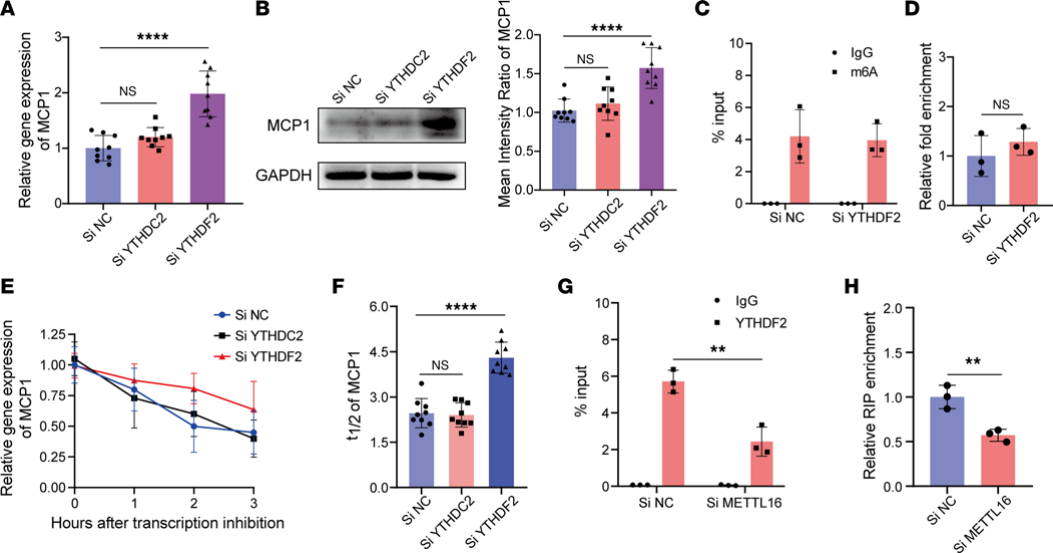
METTL16 accelerates *MCP1* mRNA degradation through YTHDF2. (**A**) Relative *MCP1* mRNA expression in siNC-, siYTHDF2-, or siYTHDC2-treated MSCs (*n* = 9). (**B**) Representative blot images and mean intensity ratio of MCP1 in siNC-, siYTHDF2-, or siYTHDC2-treated MSCs (*n* = 9). (**C** and **D**) m^6^A modification level of *MCP1* mRNA in siNC- or siYTHDF2-treated MSCs (*n* = 3). (**E**) Degradation curves of *MCP1* mRNA in siNC- or siYTHDF2-treated MSCs (*n* = 9). (**F**) The *t*_1/2_ analysis of *MCP1* mRNA in siNC- or siYTHDF2-treated MSCs (*n* = 9). (**G** and **H**) YTHDF2 RIP-qPCR analysis of *MCP1* mRNA in siNC- or siMETTL16-treated MSCs (*n* = 9). Data are presented as the mean ± SD. Two-tailed Student’s *t* test was performed in panels **C**, **D**, **G**, and **H** and 1-way ANOVA followed by Bonferroni’s test was performed in **A**, **B**, and **F**. **P < 0.01; ****P < 0.0001. NS, not significant; MSCs, mesenchymal stem cells; MCP1, monocyte chemoattractant protein 1; siNC, control siRNA; siMETTL16, siRNA for METTL16; siYTHDF2, siRNA for YTHDF2; siYTHDC2, siRNA for YTHDC2.

To investigate how m^6^A modification affects *MCP1* mRNA degradation, we further knocked down YTHDC2 or YTHDF2, which are both key RNA m^6^A readers responsible for m^6^A-mediated mRNA destabilization, in MSCs (19, 20). The knockdown efficiency of siYTHDC2 and siYTHDF2 was confirmed at both the mRNA and protein levels. Based on the qPCR and Western blot results, siYTHDC2-1 and siYTHDF2-1 were chosen for subsequent experiments (Supplemental Figure 3, E and F). After knockdown of YTHDC2 or YTHDF2, the data showed that both at the mRNA and protein levels, YTHDF2 knockdown significantly enhanced MCP1 expression compared with that of the control MSCs, while there was no significant difference between the YTHDC2-knockdown and control groups (Figure 4, A and B). To eliminate the possibility that YTHDF2 knockdown might affect the m^6^A level on *MCP1* mRNA, we quantified the m^6^A modification on *MCP1* mRNA when YTHDF2 was knocked down in MSCs using the m^6^A RIP-qPCR method. The results showed that there was no significant difference between the YTHDF2-knockdown and control MSCs (Figure 4, C and D). We further investigated the half-life of *MCP1* mRNA when YTHDC2 or YTHDF2 was knocked down in MSCs. The results showed that knocking down YTHDF2 enhanced *MCP1* mRNA stabilization compared with that of the control group, while knocking down YTHDC2 did not affect the stabilization of *MCP1* mRNA (Figure 4, E and F). Next, we analyzed the interaction between YTHDF2 and the m^6^A modification on *MCP1* using the RIP-qPCR method. The data showed that *MCP1* RNA could be immunoprecipitated by an anti-YTHDF2 antibody, and *MCP1* mRNA enrichment in the METTL16-knockdown MSCs was significantly lower than that in the control MSCs (Figure 4, G and H). Collectively, these data indicated that *MCP1* was a target of YTHDF2 rather than YTHDC2.

### YTHDF2 recognizes specific m^6^A modification sites in the MCP1 CDS region.

**Figure 5:**
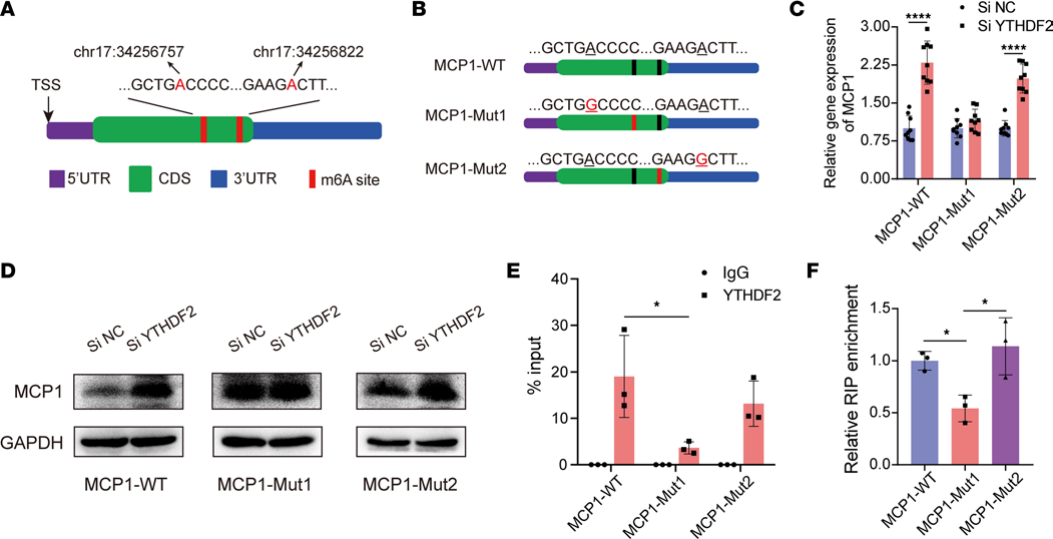
YTHDF2 recognizes specific m^6^A modification sites in the *MCP1* CDS region. (**A**) Schematic representation of m^6^A sites of *MCP1* mRNA in the CDS region. (**B**) Schematic representation of mutated m^6^A sites of *MCP1* mRNA in the pCDNA3.1 vector. Adenines in chr17:34256757 (MCP1-Mut1) and chr17:34256822 (MCP1-Mut2) were mutated to guanine. (**C**) pCDNA3.1 vectors expressing the MCP1 mutant were transfected into 293T cells treated with siNC or siYTHDF2, and the relative *MCP1* mRNA expression was quantified by qPCR (*n* = 9). (**D**) Representative blot images of MCP1 in siNC- or siYTHDF2-treated 293T cells transfected with MCP1-mutant pCDNA3.1 vectors (*n* = 9). (**E** and **F**) YTHDF2 RIP-qPCR analysis of *MCP1* mRNA in the MSCs (*n* = 9) transfected with MCP1-mutant pCDNA3.1 vectors. Data are presented as the mean ± SD. Two-tailed Student’s *t* test was performed in panels **C** and **E** and 1-way ANOVA followed by Bonferroni’s test was performed in **F**. *P < 0.05; ****P < 0.0001. NS, not significant; MSCs, mesenchymal stem cells; MCP1, monocyte chemoattractant protein 1; siNC, control siRNA; siYTHDF2, siRNA for YTHDF2.

As a m^6^A modification reader, YTHDF2 functions by recognizing specific m^6^A sites on mRNA. To verify the specific m^6^A sites on *MCP1* mRNA, we first searched m^6^A modification records from the m6AVar database (21). The results showed that m^6^A sites with high confidence were located in chr17:34256757 and chr17:34256822, which were both in the *MCP1* CDS region (Figure 5A). Then, we mutated the potential m^6^A sites in chr17:34256757 (MCP1-mut1) or chr17:34256822 (MCP1-mut2) and established expression constructs of wild-type MCP1 (MCP1-WT) and these 2 mutants in the pcDNA3.1 vector (Figure 5B). The results showed that the expression levels of MCP1-WT and MCP1-mut2 were enhanced both at the mRNA and protein levels in the YTHDF2-knockdown 293T cells, while the expression of MCP1-mut1 showed no significant differences between the normal and YTHDF2-knockdown 293T cells (Figure 5, C and D). To further confirm the m^6^A sites recognized by YTHDF2, we performed a RIP-qPCR experiment using an anti-YTHDF2 antibody. The qPCR results showed that YTHDF2-enriched *MCP1* was decreased significantly in the MSCs transfected with MCP1-mut1 compared with the MCP1-WT and MCP1-mut2 expression constructs (Figure 5, E and F). Together, these data indicated that YTHDF2 is responsible for recognizing specific m^6^A modification sites in the *MCP1* CDS region.

### Inhibiting METTL16 expression in MSCs strengthens their monocyte recruitment capacity in vivo.

**Figure 6:**
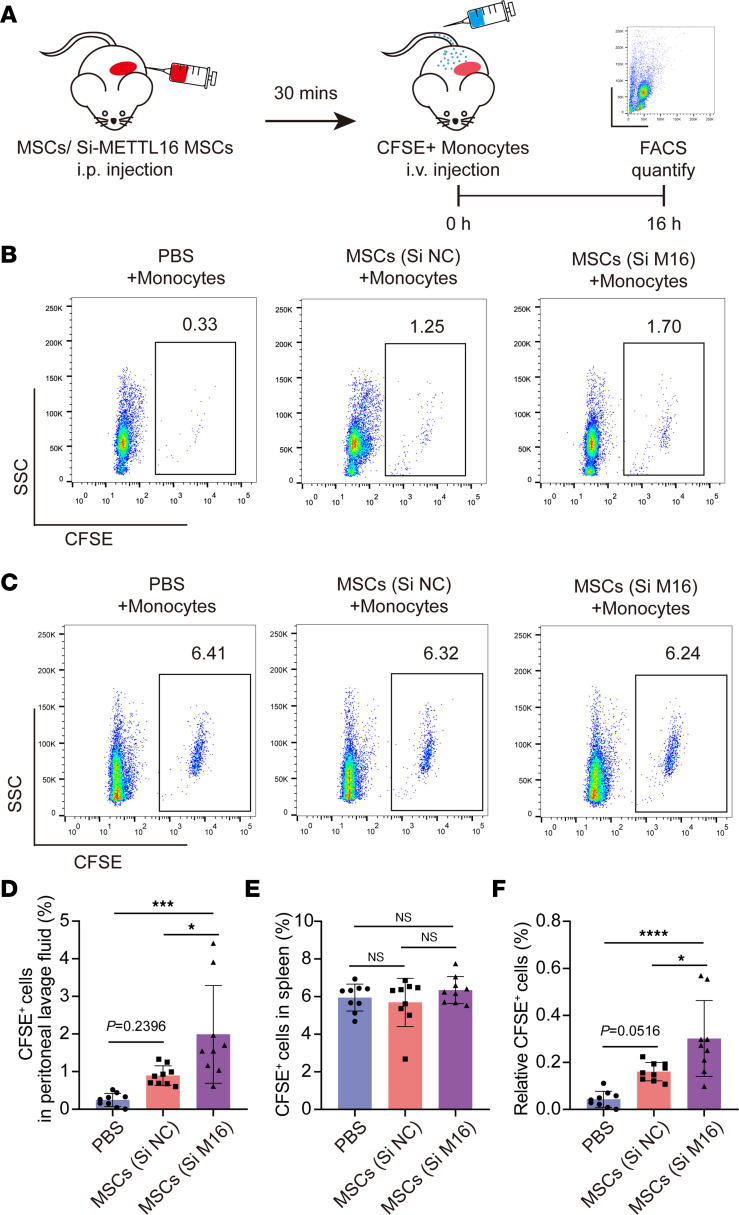
Inhibiting METTL16 expression in MSCs strengthens their monocyte recruitment capacity in vivo. (**A**) Schematic representation of the in vivo monocyte migration assay. (**B**) Representative flow cytometry histograms of CFSE-labeled monocytes in peritoneal lavage fluids of NOD/SCID mice. (**C**) Representative flow cytometry histograms of CFSE-labeled monocytes in the spleens of NOD/SCID mice. (**D**) Flow cytometry analysis of the percentage of CFSE-positive monocytes in peritoneal lavage fluids (*n* = 9). (**E**) Flow cytometry analysis of the percentage of CFSE-positive monocytes in the spleen (*n* = 9). (**F**) Relative CFSE-positive monocytes in peritoneal lavage fluids normalized to CFSE-positive cells in spleen (*n* = 9). Data are presented as the mean ± SD. One-way ANOVA followed by Bonferroni’s test was performed in **D**–**F**. *P < 0.05; ***P <0.001; ****P < 0.0001. NS, not significant; MSCs, mesenchymal stem cells.

We used NOD/SCID mice, which have impaired T and B cell lymphocyte development, to study the potential effects of METTL16 on MSC monocyte recruitment in vivo (Figure 6A). MSCs with METTL16 knockdown or negative control were injected into the abdominal cavity. The recruited monocytes in peritoneal lavage fluid could be detected and analyzed by CFSE labeling through flow cytometry. The results showed that both the METTL16-modified and normal MSCs possessed a strong monocyte recruitment capacity, while the METTL16-knockdown MSCs showed an enhanced recruitment capacity compared with the normal MSCs (Figure 6, B, D, and F). To balance the amounts of monocytes injected into each mouse, we collected the spleen of each mouse, and the percentages of CFSE-labeled monocytes were also analyzed by flow cytometry. The data showed that there were no significant differences in the amounts of recruited monocytes in the spleen among these 3 groups (Figure 6, C and E). Collectively, these data suggest that METTL16 is involved in regulating the monocyte recruitment capacity of MSCs in vivo.

## Discussion

Monocyte recruitment through MCP1 secretion is a critical aspect of the immunomodulatory function of MSCs. In this study, we found that the m^6^A methyltransferase METTL16 could negatively regulate MCP1 expression in bone marrow MSCs. Further investigation revealed that the mechanism was related to YTHDF2-mediated *MCP1* mRNA degradation. In addition, knocking down METTL16 enhanced the ability of MSCs to recruit monocytes in vivo.

MCP1-mediated monocyte infiltration is essential for many biological processes, including wound healing (9), cartilage regeneration (22), antibacterial defense (23), and gut injury protection (10). Dysfunction of MCP1 expression has been reported as an important pathophysiological mechanism in some diseases. By recruiting monocytes into the joints of individuals with osteoarthritis, increased MCP1 expression could propagate local inflammation and tissue damage and further contribute to the progression of osteoarthritis (24). In another case, Murugan et al. (25) reported that traumatic brain injury–induced monocyte infiltration was partly mediated by MCP1 signaling, and targeting MCP1 signaling could reduce the outcomes of behavioral deficits after brain injury. MSCs are one of the main sources of MCP1, and dysfunctional MCP1 secretion by MSCs can lead to pathological conditions. Pasquier et al. (26) reported that MSC-secreted MCP1 could protect ovarian cancer cells from chemotherapy, thus increasing the possibility of recurrence of ovarian cancer. Moreover, in a previous study, we found that enhanced MCP1 secretion by MSCs triggered monocyte infiltration into ossification sites and led to chronic inflammation in ankylosing spondylitis. Therefore, for both physiological and pathological conditions, it is important to investigate the mechanism by which MSCs secrete MCP1 to attract monocytes.

m^6^A is an internal modification and one of most prevalent posttranscriptional modifications in mRNAs (27). m^6^A modifications affect the splicing, degradation, and transport of mRNAs and participate in many biological processes, such as cell differentiation, immune response, and cancer development (28, 29). Recently, the importance of m^6^A modifications in regulating cell differentiation and the immunomodulatory function of MSCs has been reported. Chen et al. (30) reviewed the function and mechanisms of m^6^A modifications of MSCs to regulate bone biology and osteoporosis development and suggested m^6^A modifications as potential targets to prevent or treat osteoporosis. In a previous study, we found that m^6^A modifications play a crucial role in MSC osteogenic differentiation and participate in bone mass regulation in mice (31). In this study, we found that the m^6^A modification level of *MCP1* in MSCs was significantly upregulated but then gradually decreased when the cells were cocultured with monocytes for 0 to 36 hours. Moreover, a negative correlation was observed between MCP1 and the m^6^A methylase METTL16 expression. These results first demonstrated the effect of m^6^A modification on MCP1 expression in MSCs and then indicated the critical role of METTL16 in this process.

The m^6^A modification of mRNA is a reversible and dynamic process involving various m^6^A methylases, including METTL3, METTL14, and METTL16, and demethylases, including FTO and ALKBH5. Wu et al. (32) reported that METTL3 is essential for MSCs in fate decisions and bone formation, and knockout of METTL3 could induce the pathological features of osteoporosis in mice. By regulating the expression of key genes, METTL14 also has been reported to have functional roles in some diseases, such as steroid-associated osteonecrosis (33). In contrast to m^6^A methylases, m^6^A demethylases usually oppositely regulate m^6^A modification on some mRNAs. Song et al. (34) reported that METTL3 and ALKBH5 could oppositely regulate the m^6^A modification of *TFEB* mRNA and dictate the fate decision of cardiomyocytes in ischemic heart disease. In this study, for the first time to our knowledge, we demonstrated that METTL16 is an important effector for MSCs to regulate MCP1 expression and subsequent monocyte recruitment. Recently, the RNA-binding protein tristetraprolin (TTP) was reported to increase METTL14 expression and inhibit MCP1 expression through the m^6^A modification in the human liver cell line HL7702 (17). In our study, however, no significant differences were observed in other m^6^A-related proteins (METTL3, METTL14, FTO, and ALKBH5) of MSCs when cocultured with monocytes, and inhibiting these proteins showed no effects on the MCP1 expression of MSCs. Moreover, several posttranscriptional regulatory factors, including TTP, were shown to contribute to regulating MCP1 expression. The RNA-binding protein HuR positively regulated MCP1 expression in epithelial cells by affecting mRNA stability (35). IGFBP2, another RNA-binding protein, also increased MCP1 expression in human stromal fibroblasts by extending the mRNA half-life (36). Moreover, inhibiting these proteins showed no effects on the MCP1 expression of MSCs (data not shown). This discrepancy may, on the one hand, result from the different cells and, on the other hand, be caused by the difference in stimulating factors in the two studies.

Studies have revealed several mechanisms by which m^6^A methylases regulate gene expression, including transcription, RNA splicing, and mRNA degradation (37). Pendleton et al. (38) reported that METTL16 could regulate human MAT2A expression by promoting its RNA splicing. Furthermore, Su et al. showed that METTL16 could promote gene translation through an m^6^A-independent manner (39). In our study, we found that knocking down METTL16 in MSCs did not affect the transcriptional activity of *MCP1* mRNA or the splicing of *MCP1* RNA. In addition, knocking down METTL16 expression did not alter the translational efficiency of *MCP1* mRNA. However, knocking down METTL16 decreased *MCP1* mRNA degradation in MSCs. Thus, we speculated that METTL16 mostly affects MCP1 expression by regulating the degradation of mRNA but not the transcription and splicing of MCP1. m^6^A-regulated mRNA degradation is mainly mediated by YTHDF2 and YTHDC2 (40). We observed that knocking down YTHDF2 enhanced MCP1 expression and mRNA stability, and we further demonstrated the interaction between *MCP1* mRNA and YTHDF2. This result was also demonstrated by a recent study mentioned above (17), in which knocking down YTHDF2 increased the expression level of *MCP1* mRNA, and overexpressing YTHDF2 decreased *MCP1* mRNA stability. Interestingly, in this process, we unexpectedly found that increasing METTL16 expression indeed increased the m^6^A content on *MCP1* mRNA but did not obviously affect *MCP1* mRNA degradation in MSCs. However, *MCP1* mRNA stability increased significantly in the context of METTL16, and YTHDF2 was knocked down. We speculated that under normal culture conditions, the interaction of *MCP1* mRNA and YTHDF2 in MSCs has already reached a steady state so that further increasing METTL16 expression cannot continually enhance this interaction. However, this speculation and detailed mechanisms require further analysis.

The crystal structures of METTL3, METTL14, and METTL16 are different, so they have different substrate preferences when directly recognizing mRNA sequences. According to a previous study, METTL3 and METTL14 usually recognize single-stranded RNA with a RRACH sequence (base R is A or G; H is A, C, or U), while METTL16 usually prefers structured RNA with a UACAGAGAA sequence (38). In our study, we did not find an obvious UACAGAGAA sequence or specific RNA structure in the region of the predicted m^6^A site on *MCP1* mRNA. In our study, an ATGACC site at chr17:34256757, the CDS region of *MCP1*, was shown to be recognized by YTHDF2. This result indicated that METTL16 may bind to the RRACH sequence of *MCP1* in MSCs. The detailed binding mechanism as well as the binding structure remain to be investigated in the future.

Given the important role of m^6^A in regulating gene expression, many studies have investigated its therapeutic potential in different diseases (37, 41). The m^6^A methylase, demethylase, and readers all participated in regulating gene expression, so these enzymes all could be potential targets once their substrates are identified. Some small activators as well as inhibitors of METTL3, FTO, and YTHDF1–3 were designed to treat different cancers both in vitro and in mouse models (42). Some inhibitors of the METTTL3-METTL14 complex were investigated in phase I clinical trials of acute myeloid leukemia (43). In our study, we found that MSCs with METTL16 knockdown showed an enhanced ability to recruit monocytes in a mouse model and confirmed the potential of METTL16 to regulate MSC monocyte recruitment in vivo. These results provide basic support for the development of inhibitors that target METTL16 to enhance MSC function or treat MCP1-involved diseases, such as osteoarthritis and cancers.

In summary, we provide evidence that the m^6^A modification is involved in MCP1 expression in MSCs. METTL16 negatively regulates MCP1 expression through the m^6^A reader YTHDF2. Our data reveal a potential mechanism of regulating MCP1 expression in MSCs by modulating METTL16 expression, indicating a potential therapeutic strategy to manipulate MCP1 expression in MSCs to address or cure MCP1-involved disorders.

## Methods

### Cell isolation and culture.

The isolation and expansion of MSCs were performed as described previously (18). Briefly, bone marrow was collected from the posterior superior iliac spine of donors. Then, MSCs were isolated by density gradient centrifugation and resuspended in Dulbecco’s modified Eagle’s medium (DMEM) with 10% fetal bovine serum (FBS) and cultured at 37°C in a 5% CO_2_ atmosphere. The cells in suspension were removed, and the culture medium was replaced every 3 days. Then, cultured MSCs were digested using 0.25% trypsin and reseeded in 2 new flasks when they reached 90% confluence, and MSCs at passage 3 or 4 were used in subsequent experiments.

Peripheral blood mononuclear cells (PBMCs) were isolated via density gradient centrifugation. Then, CD14^+^ monocytes used in experiments were further isolated and purified from PBMCs using CD14 MicroBeads (Miltenyi Biotec). Monocytes were cultured in DMEM with 10% FBS at 37°C under 5% CO_2_.

HEK293T (293T) cells were purchased from the National Collection of Authenticated Cell Cultures (Shanghai, China) and cultured using high-glucose DMEM containing 10% FBS in the same atmosphere as above. For cell digestion, 0.25% trypsin containing 0.53 mM EDTA was used when cells reached 80%–90% confluence.

### RNA interference.

Two METTL3-, METTL14-, METTL16-, FTO-, ALKBH5-, YTHDF2-, and YTHDF3-specific siRNAs and siNCs were designed and synthesized by IGE Biotechnology. Detailed sequences of the siRNAs are provided in Supplemental Table 1. siRNAs were used to knock down relative gene expression in MSCs using Lipofectamine RNAi MAX (Thermo Fisher Scientific) according to the manufacturer’s protocol. The knockdown efficiencies of the siRNAs were assessed by qPCR and Western blotting after 48 hours, and the siRNA with the best efficiency was chosen for further experiments.

### Lentivirus construction and transfection.

METTL16 overexpression lentivirus (OE METTL16) and its negative control (OE NC) were constructed by and purchased from OBiO. OE METTL16 lentivirus (1 × 10^9^ TU/mL) and 5 μg/mL polybrene (OBiO) were used to infect MSCs at an MOI of 30. The culture medium was replaced after 24 hours. Further experiments were performed after another 48 hours, and the overexpression efficiency was analyzed by qPCR and Western blotting.

### Coculture of MSCs and CD14^+^ monocytes.

MSCs and monocytes were cocultured in a Transwell system using polycarbonate membrane Transwell inserts (0.4-μm pores, 6-well plate, Corning). MSCs (1 × 10^5^) in 2.6 mL of DMEM were seeded in the lower chambers, and 1 × 10^6^ monocytes were suspended in 1.5 mL of DMEM and seeded in the upper chambers.

### Monocyte migration assay.

Monocyte migration assays were performed using polycarbonate membrane Transwell inserts (5.0-μm pores, 24-well plate, Corning). A total of 3 × 10^4^ MSCs in 600 μL of DMEM or cell-free culture supernatant were seeded in the lower chambers. After adhesion, MSCs were treated for RNA interference or lentiviral transfection. Then, the culture supernatant was replaced with FBS-free culture supernatant with or without 0.5 μg/mL anti-MCP1 neutralizing antibody (MAB679, R&D Systems), and 1 × 10^6^ monocytes in 100 μL of FBS-culture supernatant were seeded in the upper chambers after staining with CFSE for 15 minutes. After 12 hours, the culture supernatant in the lower chambers was collected, and the number of monocytes in the supernatant was calculated by flow cytometry. CFSE-positive cells were regarded as migrated monocytes in the lower chambers. The molecular biological studies of MSCs were also performed after coculture with monocytes.

### qPCR.

Total RNA was extracted from MSCs using TRIzol reagent (Invitrogen), and RNA quality and concentration were measured with a Nano Photometer N60 (Implen). A total of 1000 ng of RNA was transcribed into cDNA using a Prime Script TMRT reagent kit (TaKaRa). qPCR was performed using SYBR Premix Ex Taq reagent (TaKaRa) on an Applied Biosystems 7500 Real-Time PCR System (Thermo Fisher Scientific). Primers targeting genes in the study are provided in Supplemental Table 2.

### RNA stability assay.

For the RNA stability assay performed in MSCs, cells were seeded in 12-well plates at a density of 7 × 10^4^ per well. After overnight incubation, siRNA or lentivirus specific for METTL16 was added and processed as described above. Then, actinomycin D was added to each well at a final concentration of 20 μg/mL and treated for 0, 1, 2, or 3 hours. Total RNA was extracted from MSCs, and qPCR was performed to analyze the mRNA expression of MCP1. The formulas used to analyze the half-life of target mRNA were described in a previous study and are shown briefly here (44): 

*t*_1/2_ = ln_2_/*k*_decay_, (e1)

where

*k*_decay_ = the decay rate constant. (e2)

### Polysome profiling assay.

The polysome profiling was performed according to a previous study (45). Briefly, MSCs transfected with siNC or siMETTL16 were treated with 100 μg/mL cycloheximide (MedChemExpress, HY-12320) for 10 minutes and were collected. Then, the cytoplasm was extracted, layered onto a 10%–45% sucrose gradient, and centrifuged at 222,227*g* for 2.5 hours at 4°C in an ultraspeed centrifuge (Beckman, L-100XP). The polysome fractions were collected, and the *MCP1* mRNA level was analyzed by qRT-PCR.

### MCP1 promoter activity assay.

Briefly, MSCs were seeded in 12-well plates at a density of 7 × 10^4^ per well. siMETTL16 and siNC were added and incubated for 2 days. Then, the cells were transfected with the pGL4.10 luciferase reporter vector (IGE Biotechnology) containing the –2000/+100 sequence of the *MCP1* promoter. The pRL-CMV vector containing Renilla luciferase (IGE Biotechnology) was cotransfected to normalize transfection efficiency. MCP1 transcription activity was measured using a dual-luciferase assay kit (Promega) according to the manufacturer’s instructions. The ratios between the activity of the pGL4.10 luciferase reporter (Luc) and pRL-CMV Renilla luciferase (Ruc) are displayed as the transcription activity results.

### MCP1 expression plasmid construction and transfection.

The *MCP1* mRNA sequence was generated from the NCBI reference sequence NM_002982.4. The original *MCP1* mRNA sequence was regarded as MCP1-WT. The adenine located in chr17:34256757 (MCP1-Mut1) and chr17:34256822 (MCP1-Mut2) was mutated to guanine. MCP1-WT, MCP1-Mut1, and MCP1-Mut2 were cloned into the pcDNA3.1(+) vector and generated by IGE Biotechnology. Then, 293T cells were seeded in 12-well plates at a density of 1.5 × 10^5^ cells per well. YTHDF2-specific siRNA for was added for 24 hours. Then, MCP1-WT or mutant plasmids were transfected into 293T cells using Lipofectamine 3000 reagent (Thermo Fisher Scientific). After 24 hours, total RNA or protein was extracted from 293T cells and used to analyze the expression of MCP1 using qPCR or Western blotting, respectively.

### m^6^A RIP-qPCR.

For the m^6^A RIP assay, the Magna MeRIP m^6^A Kit (Merck Millipore) was used according to the manufacturer’s protocol. Briefly, total RNA of MSCs was extracted using TRIzol reagent and fragmented. Then, prepared Protein A/G magnetic beads conjugated with anti-m^6^A antibody (ab208577, Abcam), anti-YTHDF2 (ab220163, Abcam), or IgG control were added and incubated with fragmented RNA overnight. After that, the magnetic beads were collected, and the immunoprecipitated RNA was further collected and used to analyze the m^6^A enrichment of target genes by qPCR. Nonimmunoprecipitated RNA fragments were regarded as the input control, and the formulas used in the analysis are listed as follows: 

 ΔCT_RIP_ = CT_RIP_ – CT_input_; (e3)

 %input = 2^(–ΔCT^_RIP_^)^; (e4)

 ΔCT_igG_ = CT_igG_ – CT_input_; (e5)

 ΔΔCT = ΔCT_RIP_ – ΔCT_igG_; (e6)

 fold enrichment = 2^–ΔΔCT^. (e7)

The represented relative fold enrichment was normalized to the fold enrichment of the corresponding control group.

### Western blotting.

Cell lysates of MSCs and 293T cells were collected using RIPA lysis buffer containing 1% phosphatase inhibitors and protease inhibitors. After centrifugation at 12,000*g* for 10 minutes, the supernatant was collected, and total protein concentrations were quantified using a BCA Protein Assay Kit (Thermo Fisher Scientific). Equal amounts of proteins were separated via SDS-polyacrylamide gel electrophoresis and transferred to polyvinylidene fluoride (PVDF) membranes with a pore size of 0.45 μm (Merck Millipore). PVDF membranes were blocked with 5% skim milk and incubated overnight at 4°C with 1:1000 diluted primary antibodies against MCP1 (ab214819, Abcam), METTL3 (ab195352, Abcam), METTL14 (ab220030, Abcam), METTL16 (17676S, Cell Signaling Technology), FTO (ab126605, Abcam), ALKBH5 (ab195377, Abcam), YTHDF2 (ab220163, Abcam), or YTHDC2 (ab220160, Abcam). Then, PVDF membranes were incubated with horseradish peroxidase–conjugated (HRP-conjugated) secondary antibodies (1:3000) for 1 hour at room temperature. The immunoreactivity was detected using the Immobilon Western Chemiluminescent HRP Substrate (Merck Millipore) and visualized in the UVP Chemstudio image system (Analytik Jena). The mean intensity ratio of spots was quantified by ImageJ software (NIH), and the expression of GAPDH or β-actin was used as the internal control. See complete unedited blots in Supplemental Figure 4.

### In vivo monocyte migration assay.

The in vivo monocyte migration assay was performed according to a previous study, with some modifications. Briefly, 27 male NOD/SCID mice (Gempharmatech) were separated into 3 equal groups. All mice were subcutaneously injected with 0.5 mg/kg human macrophage colony–stimulating factor (M-CSF) (PeproTech) to maintain the survival of monocytes. After 12 hours, MSCs pretreated with METTL16 siRNA or siNC were intraperitoneally injected at 5 × 10^5^ cells per mouse. Equal amounts of PBS were also injected as a negative control. After approximately 30 minutes, monocytes were prepared and stained with CFSE, and the mice were intravenously injected with 2 × 10^7^ cells per mouse. Sixteen hours after adoptive transfer, the mice were sacrificed, and the peritoneum was washed to collect the peritoneal lavage fluid for further flow cytometric analysis.

### Statistics.

The experimental data of this study were analyzed using GraphPad Prism 8.0 software, and the data are presented as the mean ± standard deviation (SD). Comparisons between 2 experimental groups were performed with 2-tailed Student’s *t* tests. One-way ANOVA followed by Bonferroni’s test was used for multiple comparisons. *P* less than 0.05 was considered statistically significant.

### Study approval.

This study was approved by the Ethics Committee of The Eighth Affiliated Hospital, Sun Yat-sen University, GuangZhou, China. All healthy donors were informed of the experimental procedure and potential risks, and the informed consent form was signed before bone marrow or peripheral blood donation. The animal experiments were approved by the Institutional Animal Care and Use Committee of Sun Yat-Sen University, GuangZhou, China.

## Author contributions

ZX, PW, YW, and HS helped design the research studies. ZZ, ZX, J Lin, PW, YW, and HS helped analyze the data. ZZ, ZX, J Lin, Z Sun, ZL, WY, YZ, GY, J Li, FY, Z Su, YC, PX, and CZ helped conduct the experiments. Order position of first authorship is based on overall intellectual contribution to interpretation of data and conducting experiments.

## Supplementary Material

Supplemental data

## Version 1 02/16/2023

In-Press Preview

## Version 2 03/22/2023

Electronic publication
