# Supplementary material for: The m6A methyltransferase METTL16 negatively regulates MCP1 expression in mesenchymal stem cells during monocyte recruitment
Source: JCI Insight. 2023 Mar 22;8(6):e162436. doi: 10.1172/jci.insight.162436 (PMC10070103; doi:10.1172/jci.insight.162436)
Supplement: Supplemental data [file jciinsight-8-162436-s117.pdf]

Supplementary Materials

Supplementary Figures

Supplementary Figure S1: The expression of METTL3, METTL14, ALKBH5 and FTO in MSCs cocultured with monocytes.

Supplementary Figure S2. Expression of MCP1 in the METTL3, METTL14, ALKBH5, and FTO knockdown MSCs.

Supplementary Figure S3. Efficiency of siRNAs and lentivirus in MSCs.

Supplementary Figure S4. Unedited Western blots.

Supplemental Tables

Supplementary Table S1: Sequence information of siRNAs.

Supplementary Table S2: Primers for qPCR.

Supplementary Figures:

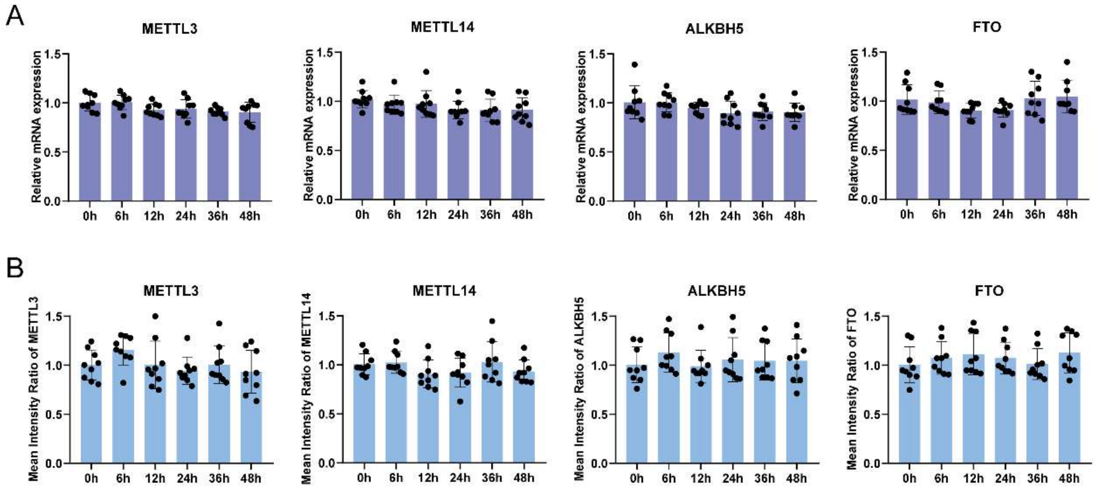

Supplementary Figure S1. The expression of METTL3, METTL14, ALKBH5 and FTO in MSCs cocultured with monocytes. A. The mRNA expression of *METTL3*, *METTL14*, *ALKBH5* and *FTO* in MSCs cultured with monocytes at different time points (n=9). **B.** The mean fluorescence intensity of METTL3, METTL14, ALKBH5 and FTO in MSCs cultured with monocytes at different time points (n=9). Data are presented as the mean ± SD.

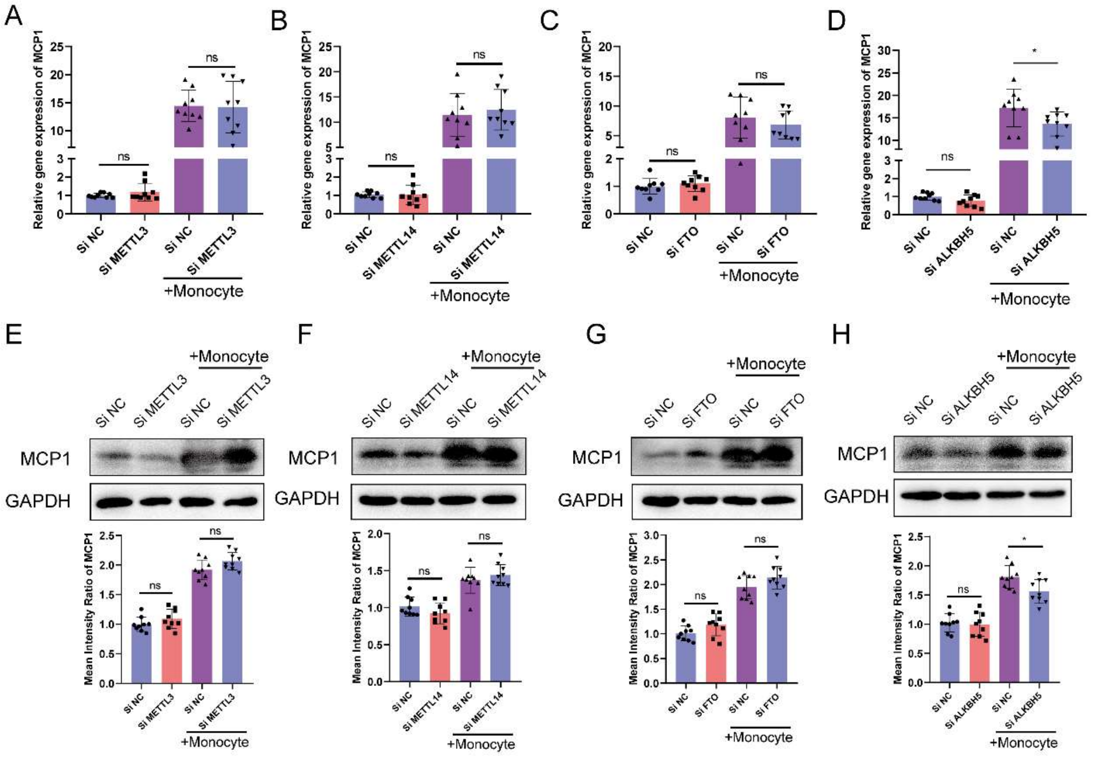

Supplementary Figure S2. Expression of MCP1 in the METTL3, METTL14, ALKBH5, and FTO knockdown MSCs. A. The mRNA expression of *MCP1* in siNC or siMETTL3 MSCs cultured with or without monocytes (n=9). **B.** The mRNA expression of *MCP1* in siNC or siMETTL14 MSCs cultured with or without monocytes (n=9). **C.** The mRNA expression of *MCP1* in siNC or siFTO MSCs cultured with or without monocytes (n=9). **D.** The mRNA expression of *MCP1* in siNC or siALKBH5 MSCs cultured with or without monocytes (n=9). **E.** Representative blot images and mean fluorescence intensity of MCP1 in siNC or siMETTL3 MSCs cultured with or without monocytes (n=9). **F.** Representative blot images and mean fluorescence intensity of MCP1 in siNC or siMETTL14 MSCs cultured with or without monocytes (n=9). **G.** Representative blot images and mean fluorescence intensity of MCP1 in siNC or siFTO MSCs cultured with or without monocytes (n=9). **H.** Representative blot images and mean fluorescence intensity of MCP1 in siNC or siALKBH5 MSCs

cultured with or without monocytes (n=9). Data are presented as the mean ± SD. ns indicates no statistical significance, and * indicates $p<0.05$.

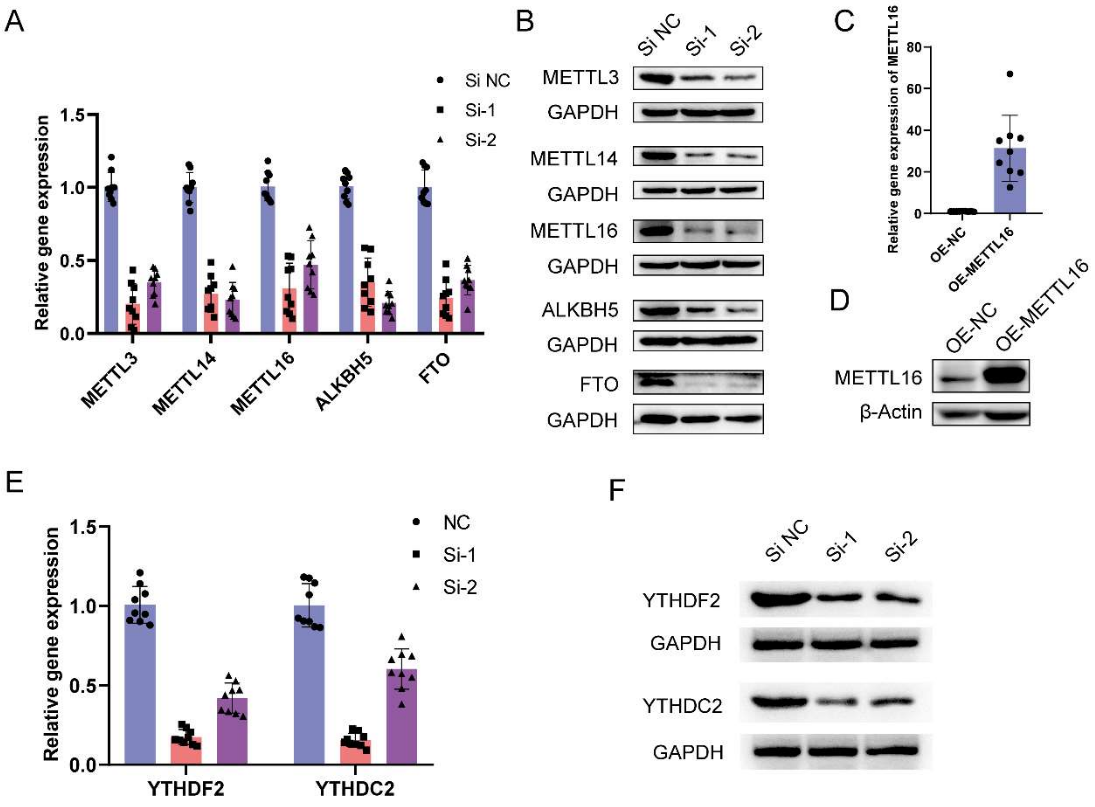

Supplementary Figure S3. Efficiency of siRNAs and lentivirus in MSCs. A. The mRNA expression of METTL3, METTL14, METTL16, ALKBH5 and FTO in the corresponding siRNA-treated MSCs (n=9). **B.** Representative blot images of METTL3, METTL14, METTL16, ALKBH5 and FTO in the corresponding siRNA-treated MSCs (n=9). **C.** The mRNA expression of *METTL16* in the lentivirus METTL16-treated MSCs (n=9). **D.** Representative blot images of METTL16 in the lentivirus METTL16-treated MSCs (n=9). **E.** The mRNA expression of *YTHDF2* and *YTHDC2* in the corresponding siRNA-treated MSCs (n=9). **F.** Representative blot images of YTHDF2 and YTHDC2 in the corresponding siRNA-treated MSCs (n=9). Data are presented as the mean ± SD.

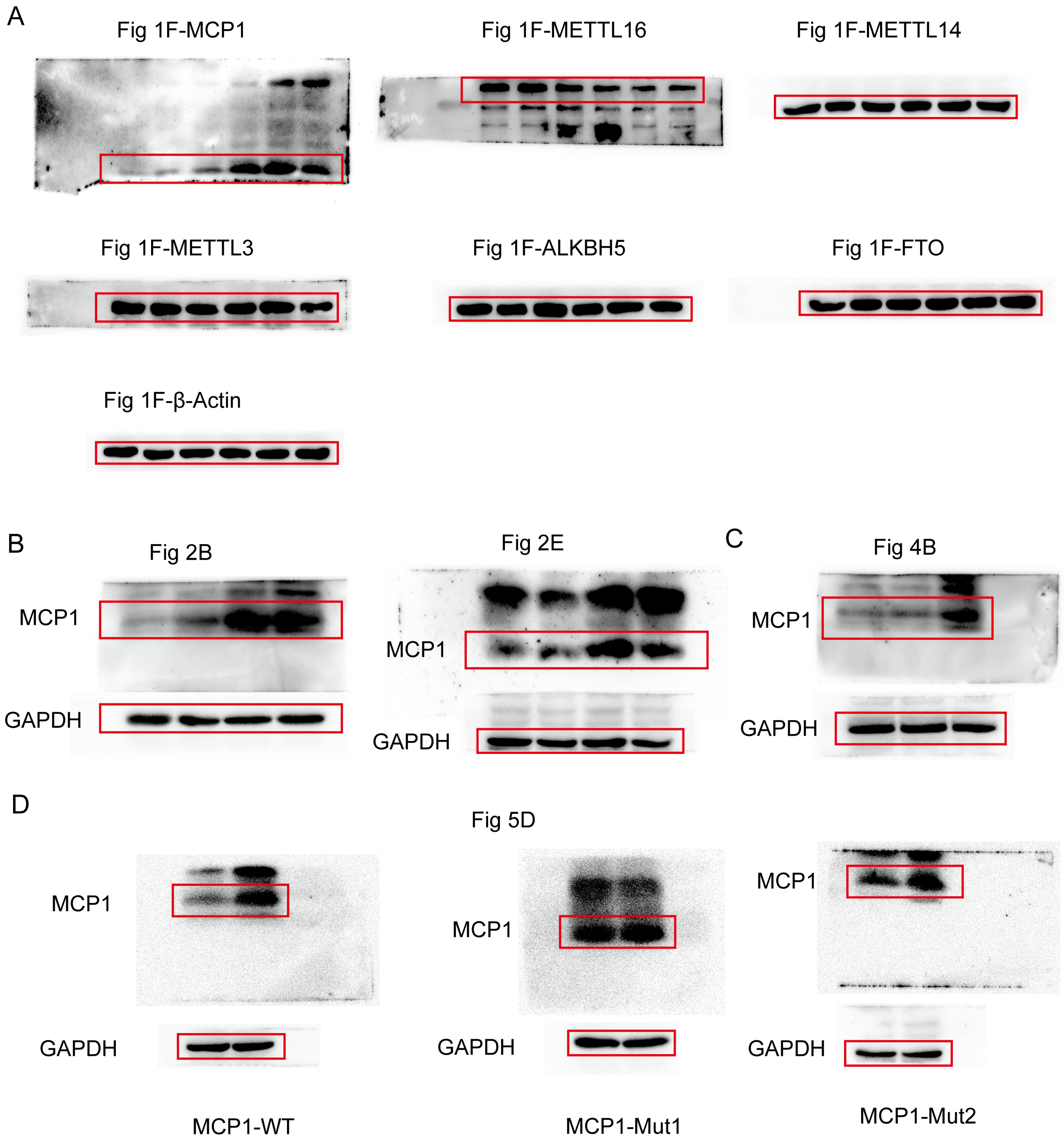

Supplementary Figure S4. Unedited Western blots.

Supplementary Tables:

Supplemental Table S1 Sequence information of siRNAs

	Sense (5'-3')	Antisense (5'-3')
Si-NC	UUCUCCGAACGUGUCACGUTT	ACGUGACACGUUCGGAGAATT
METTL3-si-1	GGGCCCAAGUGCAAGAAUUTT	AAUUCUUGCACUUGGGCCCTT
METTL3-si-2	CCUGCAAGUAUGUUCACUATT	UAGUGAACAUACUUGCAGGTT
METTL14-si-1	GGAUGAAGGAGAGACAGAUTT	AUCUGUCUCUCCUUCAUCCTT
METTL14-si-2	CCUGGGAAGACUAAGACUUTT	AAGUCUUAGUCUUCCCAGGTT
METTL16-si-1	CCAUGACAGUCUACAACUUTT	AAGUUGUAGACUGUCAUGGTT
METTL16-si-2	CCUUGAGACUCAACUAUAUTT	AUAUAGUUGAGUCUCAAGGTT
FTO-si-1	CAGGAACCUUGGAUUAUAUTT	AUAUAAUCCAAGGUUCCUGTT
FTO-si-2	GUGGCAGUGUACAGUUAUATT	UAUAACUGUACACUGCCACTT
ALKBH5-si-1	GACUGUGCUCAGUGGAUAUTT	AUAUCCACUGAGCACAGUCTT
ALKBH5-si-2	GCUUCAGCUCUGAGAACUATT	UAGUUCUCAGAGCUGAAGCTT
YTHDF2-si-1	GGUGAAGCUGCUUGGUCUATT	UAGACCAAGCAGCUUCACCTT
YTHDF2-si-2	GCACAGAAGUUGCAAGCAATT	UUGCUUGCAACUUCUGUGCTT
YTHDC2-si-1	GCGACUCAACAAUGGCAUATT	UAUGCCAUUGUUGAGUCGCTT
YTHDC2-si-2	GGAUUUGAUCAUGCAUCUUTT	AAGAUGCAUGAUCAAAUCCTT

Supplemental Table S2 Primers for qPCR

Gene	Forward primer (5'-3')	Reverse primer (5'-3')
MCP1	CAGCCAGATGCAATCAATGCC	TGGAATCCTGAACCCACTTCT
METTL3	ATTTTCCGGTTAGCCTTCGGG	TGGATTCCGTAGATCCAAGTGC
METTL14	GAACACAGAGCTTAAATCCCCA	TGTCAGCTAAACCTACATCCCTG
METTL16	ACTTTTGCATGTGCAACCCTC	ACGTTACTTTGGGAACCCCTT
FTO	GAATTCTATCAGCAGTGGCAGC	GGATGCGAGATACCGGAGTG
ALKBH5	CGGCGAAGGCTACACTTACG	CCACCAGCTTTTGGATCACCA
Pre-MCP1	GCTTCCAGAGACGGTGACTC	CCAGATGATCAGCACCAGGG
YTHDF2	GAGACCAAAAGGTCAAGGAAACA	GCAGTATATGCATTATTGGGCCTTG
YTHDC2	CGATACGGGGACCAGAGAGA	GGTCATCATTGCATGAGCTGTT
GAPDH	GGAGCGAGATCCCTCCAAAAT	GGCTGTTGTCATACTTCTCATGG
